# Experience of a Tertiary Hospital in Singapore with Management of a Dual Outbreak of COVID-19 and Dengue

**DOI:** 10.4269/ajtmh.20-0703

**Published:** 2020-09-28

**Authors:** Liang En Wee, Benjamin Pei Zhi Cherng, Edwin Philip Conceicao, Kenneth Choon-Meng Goh, Wei Yee Wan, Kwan Ki Karrie Ko, May Kyawt Aung, Xiang Ying Jean Sim, Limin Wijaya, Moi Lin Ling, Indumathi Venkatachalam

**Affiliations:** 1 Singhealth Infectious Diseases Residency, Singapore, Singapore;; 2 Department of Infectious Diseases, Singapore General Hospital, Singapore, Singapore;; 3 Department of Infection Prevention and Epidemiology, Singapore General Hospital, Singapore, Singapore;; 4 Department of Microbiology, Singapore General Hospital, Singapore, Singapore;; 5 Department of Molecular Pathology, Singapore General Hospital, Singapore, Singapore

## Abstract

During the COVID-19 pandemic, distinguishing dengue from cases of COVID-19 in endemic areas can be difficult. In a tertiary hospital contending with COVID-19 during a dengue epidemic, a triage strategy of routine COVID-19 testing for febrile patients with viral prodromes was used. All febrile patients with viral prodromes and no epidemiologic risk for COVID-19 were first admitted to a designated ward for COVID-19 testing, where enhanced personal protective equipment was used by healthcare workers until COVID-19 was ruled out. From January to May 2020, 11,086 admissions were screened for COVID-19; 868 cases of COVID-19 were diagnosed in our institution, along with 380 cases of dengue. Only 8.5% (943/11,086) of suspected COVID-19 cases were concurrently tested for dengue serology due to a compatible overlapping clinical syndrome, and dengue was established as an alternative diagnosis in 2% (207/10,218) of suspected COVID-19 cases that tested negative. There were eight COVID-19 cases with likely false-positive dengue serology and one probable COVID-19/dengue coinfection. From April to May 2020, 251 admissions presenting as viral prodromes with no respiratory symptoms were screened; of those, 15 cases had COVID-19, and 2/15 had false-positive dengue IgM. Epidemiology investigations showed no healthcare-associated transmission. In a dengue epidemic season coinciding with a COVID-19 pandemic, dengue was established as an alternative diagnosis in a minority of COVID-19 suspects, likely due to early availability of basic diagnostics. Routine screening of patients with viral prodromes during a dual outbreak of COVID-19 and dengue enabled containment of COVID-19 cases masquerading as dengue with false-positive IgM.

## INTRODUCTION

During the COVID-19 pandemic, in tropical areas with a high prevalence of arboviral diseases, distinguishing tropical infectious diseases from cases of COVID-19 can be difficult because of overlapping clinical presentations.^1,2^ Fever and nonspecific symptoms in early COVID-19 may be difficult to be distinguished from diseases such as dengue and malaria, as respiratory signs may be absent or manifest later on in the course of disease.^3^ During the previous outbreak of SARS in 2003, similar difficulties were encountered in the evaluation of patients presenting with undifferentiated fever, as dengue fever was endemic in many of the countries that faced large SARS outbreaks caused by a then-novel respiratory pathogen.^4^ This poses significant implications for infection prevention and control, as unsuspected cases of COVID-19 masquerading as undifferentiated fever^3^ may be initially managed outside of isolation areas without appropriate precautions, resulting in healthcare-associated transmission.^5^ In addition, establishment of either a diagnosis of COVID-19 or dengue fever does not entirely preclude the other, as there is a risk of coinfection in dengue-endemic areas.^6^ Coinfection or misdiagnosis is also possible in international travelers who may be exposed to both, given that dengue is a frequent diagnosis in international travelers.^7^ Furthermore, there have been isolated case reports of false-positive dengue serology with rapid diagnostic tests (RDTs) in cases of COVID-19, making it difficult to distinguish the two entities.^3,8^ Rapid serological tests play a key role in dengue diagnostics,^9^ especially in low-resource settings where resource-intensive laboratory tests such as reverse transcription–PCR (RT-PCR) and ELISA may not be routinely available.^10^ Thus, in healthcare systems facing twin outbreaks of both COVID-19 and arboviral diseases such as dengue, pathways for the care of patients presenting with undifferentiated viral fever may need to be reworked to minimize the potential of healthcare-associated transmission from a previously unsuspected case of COVID-19.^3^ The extent of cross-reactivity and false positivity in dengue RDTs and COVID-19 also needs to be clarified, given the significant reliance on RDTs for the diagnosis of dengue on a day-to-day basis.

In Singapore, a Southeast Asian tropical city-state, the first imported case of COVID-19 was reported in end-January 2020, followed by the first documented case of local transmission in early February 2020.^11^ By end-February 2020, most cases were locally transmitted.^11^ As of end-May 2020, there were almost 36,000 cases of COVID-19 reported in Singapore, with most cases occurring among migrant workers living in communal dormitories.^12^ At the same time, dengue is endemic in tropical Singapore. Periodic switches in the prevailing dengue serotype are associated with dengue epidemics during the peak dengue season^13^ and account for a significant burden in terms of hospitalizations.^14^ In the week ending May 30, 2020, Singapore reported a record weekly high of 733 dengue cases, a peak not seen since 2013, the largest outbreak year in recent history.^15^ Locally, there have been sporadic case reports of COVID-19 initially diagnosed as dengue due to false-positive dengue serology on RDTs.^8^ In these cases, COVID-19 was originally not suspected during the initial encounter because of a paucity of respiratory symptoms and a clinical syndrome initially consistent with dengue (fever and thrombocytopenia); the diagnosis of COVID-19 was delayed and only established on re-presentation and clinical deterioration, with infection prevention and control implications and potential risk of healthcare-associated transmission.^8^ At our institution, the largest tertiary hospital in Singapore, during the COVID-19 outbreak, from early February 2020, all admissions were systematically screened for respiratory symptoms and tested for SARS-CoV-2.^16^ From April 2020 onward, recognizing that COVID-19 could potentially manifest as undifferentiated viral fever with minimal respiratory symptoms, all patients with symptoms, signs, and laboratory results suggestive of a viral prodrome were also admitted to dedicated areas where COVID-19 was first ruled out. We describe our institution’s experience with the challenge of a dual outbreak of COVID-19 and dengue, focusing on the degree of overlap between possible clinical presentations of COVID-19 and dengue fever, the potential confounding of a diagnosis of COVID-19 by a false-positive dengue RDT, and containment of hitherto unsuspected COVID-19 presenting as undifferentiated viral fever outside of the isolation ward (IW).

## MATERIALS AND METHODS

### Institutional setting and study period.

The Singapore General Hospital is the largest public tertiary hospital in Singapore, with 1,785 beds. From January 2020 to May 2020, when vigilance was being maintained for potential cases of COVID-19 admitting to our institution, we evaluated the proportion of suspected COVID-19 cases (defined as a patient in which COVID-19 testing was performed) that also required a concurrent dengue diagnostic test, as well as the number of confirmed cases of dengue and COVID-19 managed in our institution. In our institution, dengue diagnostic tests were ordered at the discretion of the primary physician when a clinical syndrome potentially suggestive of dengue was encountered. A risk-stratified approach was adopted for COVID-19 screening from February 2020, in which all admissions with high-risk epidemiology (e.g., possible contact with COVID-19 cases or clusters, or travel history to areas with outbreaks of COVID-19), pulmonary infiltrates on chest imaging, or respiratory symptoms were screened for COVID-19.^16^ All patients with high-risk epidemiology for COVID-19 were admitted directly to our institution’s IW, which was equipped with negative-pressure airborne isolation rooms; patients without epidemiology risk factors were admitted to a “respiratory surveillance ward (RSW),” where COVID-19 was tested for; patients would only be transferred out of the RSW if COVID-19 tests were negative on two consecutive occasions, performed at least 24 hours apart.^16^

### Workflow for patients presenting with undifferentiated viral prodromes during a COVID-19 outbreak.

During the COVID-19 outbreak, all patients with fever were triaged in designated “fever areas” of the emergency department (ED), where healthcare workers (HCWs) used full personal protective equipment (PPE), comprising N95 respirators, gowns, gloves, and eye protection, and infrastructural enhancements were introduced, such as partitions between patient cubicles and more frequent cleaning, to minimize the potential of exposure to an unsuspected case of COVID-19.^17^ Basic investigations including a full blood count with differentials, C-reactive protein, and chest radiograph were performed routinely for all patients presenting with fever in the ED, to aid in risk stratification. Dengue RDTs could also be ordered in the ED. The strategy was to contain patients presenting with clinical syndromes compatible with COVID-19 but without epidemiological risk factors in designated inpatient areas for COVID-19 testing. Initially, patients with respiratory symptoms were admitted to the RSW; from April 2020, given the rising number of COVID-19 and dengue cases, patients requiring admission who had concurrent undifferentiated viral prodromes (e.g., fever and a normal procalcitonin, or lymphopenia/monocytosis) and no epidemiology risk for COVID-19 were also admitted to the RSW for COVID-19 testing, even in the presence of a positive dengue RDT. In the RSW, to reduce the likelihood of healthcare-associated transmission, patients were nursed either in single rooms or cohort rooms with 2–3 patients to a room (as compared with the usual norm of 5–6 patients in open-plan cohorted cubicles in our institution’s general ward); patients were given surgical masks to wear, and social distancing was encouraged; HCWs in these wards used full personal PPE, comprising N95 respirators, gowns, gloves and eye protection when caring for these patients.

### Epidemiology investigations.

On detection of a confirmed case of COVID-19 in the RSW, the affected room or cohort cubicle was locked down. Both the confirmed case and any potentially exposed patients were transferred to the IW. Simultaneously, contact tracing was performed to identify HCWs who had come into contact with the confirmed case, and risk stratification was performed based on the duration of contact, nature of activity (e.g., aerosol-generating procedures), and PPE worn at the time of contact. Both potentially exposed patients and HCWs deemed to be at high risk of exposure based on our local Ministry of Health’s guidelines were placed under a 14-day quarantine, in which they were monitored for symptoms such as cough, dyspnea and myalgia, and twice daily temperature measurements. Exposed patients who had unresolved medical issues served out their quarantine period in the IW, whereas exposed patients who were medically fit for discharge were discharged to serve out their quarantine at home. If exposed patients and HCWs developed symptoms, swabs were sent for COVID-19 testing.

### COVID-19 testing.

Oropharyngeal specimens were taken with Dacron-tipped swabs within 24 hours of admission to the RSW or IW; if oropharyngeal sampling was not feasible, other respiratory specimens, such as sputum, nasopharyngeal swab, nasopharyngeal aspirates, or bronchoalveolar lavage, were obtained. Respiratory specimens were tested for SARS-CoV-2 RNA. This was performed by qualitative real-time RT-PCR testing. Viral RNA was first extracted from patient’s samples, and RT-PCR was performed targeting E gene and ORF1b-nsp14 for SARS-CoV-2.^18,19^ As this test is performed in-house, results are usually returned within 24 hours.

### Dengue diagnostics.

At our institution, the SD Bioline Dengue Duo (Abbott Diagnostics, Santa Clara, CA) is used as an RDT on venous blood samples taken in the ED as it can be performed on demand with a rapid turnaround time. This is a commercially available rapid immunochromatographic test that comes in a combo of two joint cassettes, one for nonstructural protein 1 (NS1) antigen (Ag) and another for IgM/IgG. The manufacturer claimed that the combined NS1 Ag and/or IgM and IgG sensitivity for their test is 94.3% from 1 to 7 days after symptom onset. However, a study found that this combined sensitivity was only 82.4% (95% CI: 76.8–87.1), with a specificity of 87.4% (95% CI: 82.8–91.2); the probability of a false-negative diagnosis would be further reduced to 14.7% (95% CI: 11.4–18.6) if SD Bioline NS1 Ag/IgM/IgG combo was negative.^9^ In the inpatient setting, dengue NS1 Ag and IgM test is performed on venous blood samples using enzyme-linked immunosorbent assay (EIA) methods, which has better sensitivity and specificity but with a longer turnaround time due to batch testing. Reverse transcription-PCR for dengue virus from blood and urine specimens is also available for additional confirmatory testing.

### Ethics approval.

As this was a descriptive study based on data collected by the IPE department as part of surveillance and outbreak management, waiver of informed consent was approved by our hospital’s institutional review board (CIRB Ref 2020/2436).

## RESULTS

From January to May 2020, a total of 868 cases of COVID-19 were diagnosed upon admission to our institution, along with 380 cases of dengue fever. A total of 11,086 admissions were screened for COVID-19, of which 7.8% (868/11,086) tested positive for COVID-19. Only a small minority (8.5%, 943/11,086) of cases screened for COVID-19 had dengue serology (either RDT or EIA) concurrently ordered by the primary physician, due to a compatible clinical syndrome overlapping with both COVID-19 and dengue. Dengue was established as the main differential diagnosis in a small minority of cases (2.0%, 207/10,218) initially admitted because of a suspicion of COVID-19, based on the serological test and presentation with a clinically compatible syndrome. Among the 868 patients with PCR-confirmed COVID-19 infection, 8.1% (70/868) had dengue serology performed before confirmation of the diagnosis of COVID-19, in search of an alternative etiology for undifferentiated viral fever. Of those, a substantial proportion (12.9%, 9/70) had a positive dengue serology. The details of the nine cases with COVID-19 infection and positive dengue serology are given in Table 1. Only one case was dengue NS1 Ag positive, but IgM negative. Given a compatible clinical syndrome (protracted thrombocytopenia and petechial rash), this case was classified as COVID-19 upper respiratory tract infection (URTI) with probable dengue coinfection. Of the remaining eight cases which were positive for dengue IgM only, in five cases, this result was deemed false positive because of the absence of compatible clinical syndrome (e.g., presence of respiratory symptoms in one case, lack of thrombocytopenia in three cases, and presence of pulmonary infiltrates in one case); hence, further laboratory testing was not indicated. In addition, four of these patients presented within 2–3 days of onset of illness, and thus the absence of the early marker of dengue infection, that is, NS1 Ag, along with the unexpected presence of IgM made these results doubtful, as generally IgM should appear later, at least 5 days into illness. In three cases, blood PCR for dengue virus was sent because of the possibility of a compatible clinical syndrome (e.g., thrombocytopenia, fever, lack of pulmonary infiltrates, and rash). All these cases were PCR negative and hence deemed to be false positive on the RDT. Of the nine cases of COVID-19 which were screened dengue positive by RDT, the majority (7/9) were admitted to the IW directly because of high-risk epidemiology (e.g., potential contact with COVID-19 cases or clusters).

**Table 1:** Cases of COVID-19 infection and positive dengue serology presenting to an acute tertiary hospital in Singapore, January 2020–May 2020 (*N* = 9)

Case number	Biodata	Comorbidities	COVID-19 epidemiology risk	Presenting symptoms	Pulmonary infiltrates on CXR	Thrombocytopenia	SARS-CoV-2 results	Dengue tests (serology sent on the day of admission)	Diagnosis
Case 1	31-yo Bangladeshi male	Nil	Stays in communal setting (dormitory), nil contact with confirmed COVID-19 cases but roommates unwell	Fever, sore throat, headache, myalgia, ageusia (D2 symptom onset)	No	Yes Platelet count: 186-> 154→135-> 139-> 109-> 176 (10^9^/L)	SARS-CoV-2 PCR (oropharyngeal swab) +ve from D1, D5, D10, D11 of presentation	Dengue NS1 +ve Dengue IgM −ve	COVID-19 URTI with probable dengue coinfection (NS1 positive)
Case 2	31-yo Bangladeshi male	Nil	Stays in communal setting (dormitory), five roommates COVID-19 +ve	Fever, headache, myalgia, dry cough (D3 symptom onset)	No	Yes Platelet count: 131-> 122→141 (10^9^/L)	SARS-CoV-2 PCR (oropharyngeal swab) +ve from D1, D5 of presentation	Dengue NS1 −ve Dengue IgM +ve	COVID-19 URTI with likely false-positive dengue IgM
Case 3	38-yo Bangladeshi male	Nil	Stays in communal setting (dormitory), nil contact with confirmed COVID-19 cases but roommates unwell	Fever, sore throat, headache, myalgia, blurring of vision (D7 symptom onset)	No	No	SARS-CoV-2 PCR (oropharyngeal swab) +ve from D1, D10, D14, D18, D22 of presentation	Dengue NS1 −ve Dengue IgM +ve	COVID-19 URTI with likely false-positive dengue IgM
Case 4	34-yo Bangladeshi male	Nil	Stays in communal setting (dormitory), nil contact with confirmed COVID-19 cases but roommates unwell	Vomiting, diarrhea, chest pain (D2 symptom onset)	No	No	SARS-CoV-2 PCR (oropharyngeal swab) +ve from D1, D5 of presentation	Dengue NS1 –ve Dengue IgM +ve	COVID-19 URTI with likely false-positive dengue IgM
Case 5	29-yo Bangladeshi male	Nil	Stays in communal setting (dormitory), 11 roommates COVID-19 +ve	Fever, headache, myalgia, cough, diarrhea (D2 symptom onset)	No	No	SARS-CoV-2 PCR (oropharyngeal swab) +ve from D1, D5, D10 of presentation	Dengue NS1 –ve Dengue IgM +ve	COVID-19 URTI with likely false-positive dengue IgM
Case 6	69-yo Chinese female	Hypertension, hyperlipidemia, hypothyroidism	Works as pharmacy assistant, nil contact with COVID-19 cases or clusters	Fever (D2 symptom onset)	Yes. Bilateral patchy ground-glass infiltrates	Yes. Mild thrombocytopenia at presentation→120 (10^9^/L), subsequently normalised	SARS-CoV-2 PCR (oropharyngeal swab) +ve from D1, D5 of presentation; SARS-CoV IgG +ve from D5 of presentation; Blood SARS-CoV-2 PCR +ve	Dengue NS1 –ve Dengue IgM +ve	COVID-19 pneumonia with likely false-positive dengue IgM
Case 7	38-yo Indian male	Nil	Stays in communal setting (dormitory), nil contact with confirmed COVID-19 cases but roommates unwell	Fever, sore throat, headache, myalgia, vomiting, diarrhea (D2 symptom onset)	No	No	SARS-CoV-2 PCR (oropharyngeal swab) +ve from D1, D10, D14, D18, D22 of presentation	Dengue NS1 –ve Dengue IgM +ve Dengue/chikungunya/zikavirus blood PCR –ve D4 illness	COVID-19 URTI with false-positive dengue IgM
Case 8	34-yo Bangladeshi male	Nil	Stays in communal setting (dormitory) but nil contact with confirmed COVID-19 cases	Fever, headache, vomiting, dysgeusia (D2 symptom onset)	No	Yes. Mild thrombocytopenia at presentation→125 (10^9^/L), subsequently normalised	SARS-CoV-2 PCR (oropharyngeal swab) +ve from D1, of presentation, −ve from D10 of presentation	Dengue NS1 –ve Dengue IgM +ve Dengue/chikungunya/zikavirus blood PCR –ve D4 illness	COVID-19 URTI with false-positive dengue IgM
Case 9	48-yo Chinese male	Nil	Works as warehouse supervisor, nil contact with COVID-19 cases or clusters	Fever, myalgia, lethargy (D7 symptom onset)	No	Yes Platelet count: 96-> 88→82-> 109-> 140 (10^9^/L)	SARS-CoV-2 PCR (oropharyngeal swab) +ve from D1, D5 of presentation	Dengue NS1 –ve Dengue IgM +ve Dengue/chikungunya/zikavirus blood PCR –ve D7 illness	COVID-19 URTI with false-positive dengue IgM

**Figure 1.**
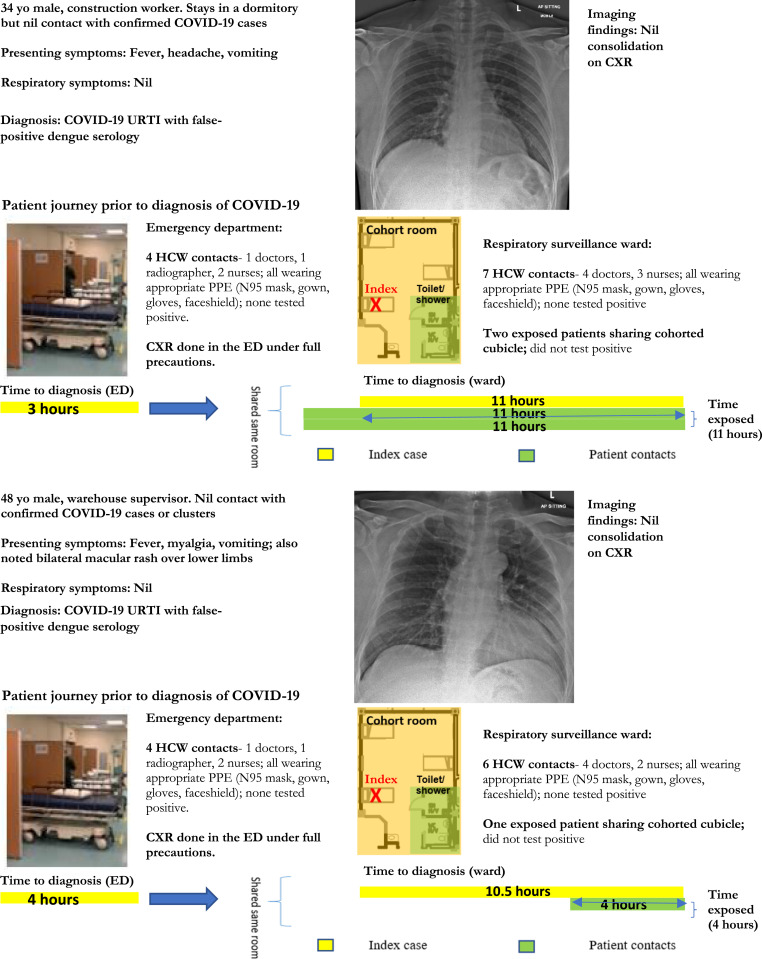
Clinical details, imaging findings, and epidemiology investigations for COVID-19 patients with false-positive dengue serology at a tertiary hospital in Singapore, during a COVID-19 outbreak (*N* = 2).

From April to May 2020, all cases of undifferentiated viral prodromes (e.g., fever and a normal procalcitonin, or lymphopenia/monocytosis) and no epidemiology risk for COVID-19 were also admitted to our institution’s RSW for COVID-19 testing, as part of a strategy to contain COVID-19 during a concurrent dengue outbreak. A total of 1,751 patients were admitted to the RSW from April to May 2020, of which 14.3% (251/1,751) presented as an undifferentiated viral prodrome with no respiratory symptoms. A total of 15 cases were found to have COVID-19 in the RSW; one-third (5/15) presented as an undifferentiated viral prodrome, with no respiratory symptoms or infiltrates on pulmonary imaging and had dengue serology RDT. Of those, two had false-positive dengue IgM in the absence of high-risk epidemiology for COVID-19. Our institution’s policy of screening patients presenting with viral prodromes and nursing such patients in a designated RSW with decreased bed density and full PPE enabled the containment of these two patients in our institution’s RSW and mitigated potential exposure (Figure 1). A total of 21 HCWs and three patients came into close contact with the two index cases of COVID-19 and false-positive dengue IgM in the RSW. However, there was no evidence of onward transmission despite intensive surveillance of exposed HCWs and patients for 21 days, likely because full PPE was used; lockdown of the wards was not required.

## DISCUSSION

Although there is concern that tropical infectious diseases such as dengue may masquerade as cases of COVID-19 and isolated case reports have appeared in the literature to illustrate this point,^2^ it appears that in practice, the potential overlap in clinical syndromes is not large, even in a dengue-endemic area. Although our institution had to contend with the emergence of a COVID-19 pandemic during a dengue epidemic season, primary physicians were only compelled to rule out dengue in less than one-tenth of patients with confirmed COVID-19 infection, and dengue fever was a main differential diagnosis in only 2% of COVID-19 suspects. Although fever, thrombocytopenia, and absence of respiratory symptoms/pulmonary infiltrates on chest imaging can manifest in COVID-19 as well as dengue, given that only ∼40% of patients with COVID-19 present with fever,^20^ one-fifth have no pulmonary infiltrates on chest imaging,^20^ and around 5–12% of patients with COVID-19 have thrombocytopenia^21^; this clinical overlap may not be commonly encountered in practice. In addition, although a petechial rash is often encountered in dengue, cutaneous manifestations have been reported in only a minority of COVID-19 cases.^22^ The rate of coinfection with COVID-19 and dengue was also low, with only one probable detected case so far in our institution.

However, our institution’s experience also demonstrates that when grappling with a COVID-19 pandemic during a dengue epidemic season, a triage strategy is necessary to detect cases of COVID-19 that may potentially be misdiagnosed as dengue. This may also be relevant in the case of international travelers^7^; although travel bans and restrictions may have lowered the risk of dual exposure, clinicians need to be aware of such a potential as travel restrictions are gradually lifted. This study also highlights that sera of COVID-19 patients can commonly cause cross-reactivity results in dengue IgM using SD Bioline Dengue Duo RDT assay. Indeed, in the existing literature, a single case of COVID-19 with likely false-positive dengue serology resulted in the inadvertent exposure of 25 HCWs and 21 patients, with confirmed patient–HCW transmission to one HCW who had attended to the patient without wearing a mask, demonstrating the risk of healthcare-associated transmission.^5^ In our institution, approximately one-third of COVID-19 cases without epidemiologic linkages presented with an isolated viral prodrome, without infiltrates on chest imaging. Before the institution of a strategy to admit all febrile patients with viral prodromes and no epidemiology risk for COVID-19 to a designated area (the RSW) for COVID-19 testing, a single case of COVID-19 who presented to our institution with a viral fever and minimal respiratory symptoms was admitted to the general ward for 19 hours, resulting in the inadvertent exposure of 20 patients and eight HCWs, although there was no evidence of onward transmission.^23^ Indeed, in that case, the rapid exclusion of dengue as an alternative diagnosis through utilization of RDTs, and persistence of the fever with no clear alternative etiology prompted the alert clinicians to consider COVID-19 and transfer the patient for testing. The concept of using “fever wards” as a triage strategy first emerged as part of efforts to contain an outbreak of SARS in 2003.^24^ In our institution, since February 2020, the strategy of containing patients presenting with clinical syndromes compatible with COVID-19 but without epidemiological risk factors in designated RSWs has successfully mitigated the risk of healthcare-associated transmission from undetected cases; to date, there have been no cases of patient–HCW transmission.^16^ In contrast to SARS, diagnostic abilities for COVID-19 were established much earlier in the current pandemic, and early rule-in or rule-out of COVID-19 is hence possible,^4^ allowing patients to be de-isolated from the “fever wards” once COVID-19 is excluded.

In healthcare systems facing overlapping epidemics of dengue and COVID-19,^25,26^ our study highlights that adherence to a strict triage algorithm to differentiate the diseases is necessary for infection prevention and control, and the possibility of coinfection, although low, needs to be excluded thoroughly.^27^ However, this strategy may not be easily emulated across all healthcare facilities universally. Admitting febrile patients with viral prodromes and no epidemiology risk for COVID-19 to a designated ward for COVID-19 testing requires various support including the availability of in-house PCR testing for COVID-19 with rapid turnaround time within 24 hours, which allowed for timely turnover of beds and an average length of stay under 48 hours in the “fever ward.”^16^ At the point of ED triage, a comprehensive set of basic diagnostics including full blood count with differentials and chest radiograph was also available to support the triage strategy and allowed for early differentiation of COVID-19 versus dengue. In resource-poor settings with limited access to supportive diagnostic tools, differentiating between these infections based on clinical signs and symptoms alone would certainly be more challenging.

The limitations of our study were as follows. Dengue IgM may be a false positive in other conditions as well, such as malaria, other flaviviruses, or autoimmune conditions with a positive rheumatoid factor.^8-10^ Establishing the diagnosis of dengue via RT-PCR is crucial to distinguish true coinfection from possible cross-reactivity. Among our COVID-19 cases with concomitant positive dengue IgM serology, only those with clinical indications were followed up with a dengue PCR blood test to exclude dengue infection. Finally, exhaustive surveillance, testing, and isolation before the return of results in designated areas with upgraded PPE might not be feasible in a healthcare system overwhelmed by an influx of COVID-19 and dengue cases, without access to adequate PPE.

In conclusion, in a tertiary hospital in a dengue-endemic country contending with the emergence of a COVID-19 pandemic during a dengue epidemic season, dengue fever was the main differential diagnosis in only a small minority of COVID-19 suspects, likely because the early availability of chest imaging and basic diagnostic testing at the point of triage enabled clinicians to distinguish between dengue and COVID-19 in most of the cases. However, for the minority of unsuspected COVID-19 cases without epidemiological risk factors and a clinical syndrome compatible with dengue (fever, thrombocytopenia, and absence of pulmonary infiltrates), potential for misdiagnosis of dengue exists due to the issue of false-positive IgM dengue serology by RDT. A triage strategy of admitting febrile patients with viral prodromes and no epidemiology risk for COVID-19 to a designated ward for COVID-19 testing over a 2-month period successfully mitigated the risk of healthcare-associated transmission from undetected cases during a dual outbreak of COVID-19 and dengue fever.
